# The Effect of Adding Transcranial Direct Current Stimulation to Hyperbaric Oxygen Therapy in Patients With Delayed Encephalopathy After Carbon Monoxide Poisoning: A Randomised Controlled Trial

**DOI:** 10.3389/fneur.2021.719765

**Published:** 2021-12-01

**Authors:** Huifang Cao, Xiaona Tan, Zibo Liu, Long Zhao, Lin Chi, Manyu Li, Chunhui Liu, Hongling Li

**Affiliations:** ^1^The Department of Rehabilitation, The Second Hospital of Hebei Medical University, Shijiazhuang, China; ^2^Department of Neurological Rehabilitation, Hebei Medical University Affiliated Children's Hospital of Hebei Province, Shijiazhuang, China; ^3^The Department of Endocrinology, The Second Hospital of Hebei Medical University, Shijiazhuang, China

**Keywords:** transcranial direct current stimulation, hyperbaric oxygen, delayed encephalopathy after carbon monoxide poisoning, cognitive function, activities of daily living

## Abstract

**Objective:** To investigate the effect of transcranial direct current stimulation (tDCS) combined with hyperbaric oxygen therapy (HBOT) in patients with delayed encephalopathy after carbon monoxide poisoning (DEACMP).

**Design:** A parallel-group, open-label randomised controlled study.

**Setting:** Hyperbaric Oxygen Therapy Room of the Second Hospital of Hebei Medical University.

**Subjects:** A total of 40 patients were recruited for the current study. Patients were randomly divided into a treatment group and a control group (20 cases/group).

**Interventions:** Control group: conventional, individualised rehabilitation therapy. Treatment group: conventional, individualised rehabilitation therapy and tDCS.

**Main Measures:** cognitive function of patients, the Barthel Index (BI).

**Results:** After treatment, significantly higher MMSE and BI scores, as well as a greater reduction in P300 latency and an increase in P300 amplitude, were observed in the treatment group compared to the control group (MMSE: 13 ± 7 vs. 9 ± 5; P300 latency: 342 ± 29 vs. 363 ± 17 ms; P300 amplitude: 7.0 ± 3.3 vs. 5.1 ± 2.7 μV; all *P* < 0.05). In both groups, however, MMSE and BI scores, in addition to P300 amplitude, were significantly improved; in contrast, there was a decrease in P300 latency in both groups after treatment compared to before treatment (all *P* < 0.05).

**Conclusion:** Combined with HBOT, tDCS can help improve cognitive function and ADL in patients with DEACMP. This combination therapy might be a helpful method to enhance the recovery of patients with DEACMP.

## Introduction

The incidence and mortality of acute carbon monoxide poisoning rank first among the occupational hazards in China (1). Acute carbon monoxide poisoning may lead to many neurophysiological and neuropathological changes, and can cause severe symptoms, including dementia, incontinence, psychosis, Parkinson's syndrome and epilepsy (2, 3). Moreover, in 3–40% of patients, it may cause delayed encephalopathy after acute carbon monoxide poisoning (DEACMP). Delayed encephalopathy after carbon monoxide poisoning was a group of neuropsychiatric symptoms mainly acute dementia in patients with carbon monoxide poisoning after recovery from rescue. It usually occurs within 2 months after acute poisoning. At present, the pathogenesis of DEACMP is not clear. DEACMP is related to many factors, such as ischemia, hypoxia, reperfusion injury, immune dysfunction, cytotoxic injury and neurotransmitter imbalance (4, 5). At present, different types of medications, hyperbaric oxygen therapy and various rehabilitation methods have been applied in the treatment of delayed encephalopathy after acute carbon monoxide poisoning. However, these treatments are difficult to control the further progress of the disease. Therefore, exploring more effective treatments is the focus of current research.

Transcranial direct current stimulation (tDCS) is a non-invasive, low-intensity, constant weak current (1–2 mA) technique that regulates excitability of cortical neurons (6). tDCS may regulate cognitive function by increasing local cerebral blood flow, promoting cerebral circulation and improving synaptic plasticity. It has been used in the treatment of sequelae of stroke, Parkinson's disease, Alzheimer's disease, epilepsy and depression (7–12). tDCS may promote cerebral circulation and improve synaptic plasticity by increasing local blood flow in the brain (13). It activates sodium, calcium dependent channels and NMDA receptor activities, depolarize or hyperpolarize neuronal membrane potential, so as to regulate neural activity and cortical excitability, act on key brain regions related to cognitive process, cause excitability changes in relevant brain regions, and improve their cognitive function (14).

The aim of this study was to investigate the efficacy of transcranial direct current stimulation combined with hyperbaric oxygen therapy in the treatment of delayed encephalopathy after acute carbon monoxide poisoning providing a reference for clinical treatment.

## Materials and Methods

### Subjects, Inclusion, and Exclusion Criteria

**Figure 1:**
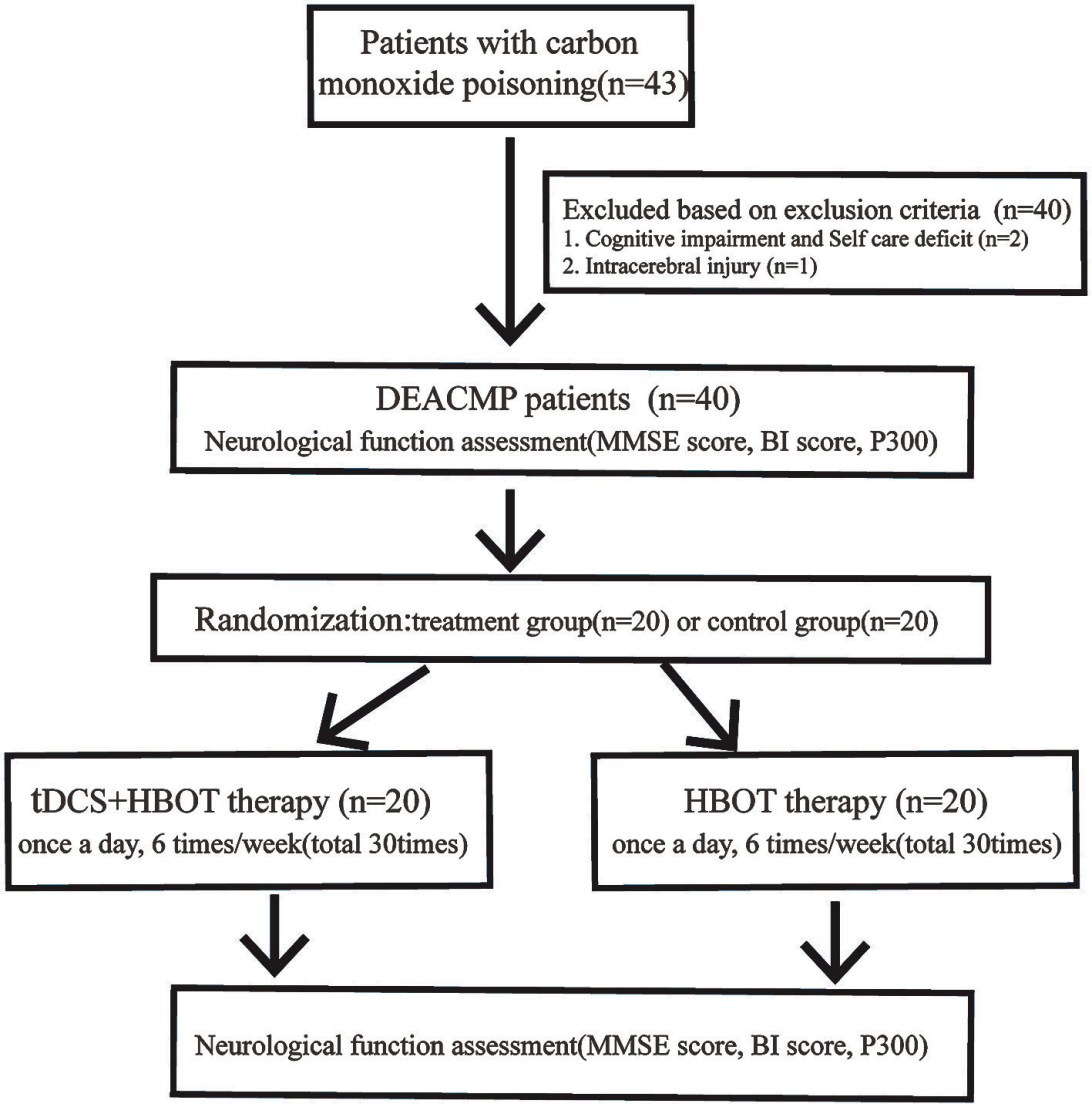
The flow diagram.

This was a parallel group, randomised controlled study. From December 2016 to February 2018, forty patients with delayed encephalopathy after acute carbon monoxide poisoning who were admitted to the Second Department of Rehabilitation Medicine (Hyperbaric Oxygen Therapy Room) of the Second Hospital of Hebei Medical University were selected for this study (Figure 1). The random allocation was referred to the random number table without blinding. This study was conducted in accordance with the Declaration of Helsinki and approved by the ethics committee of the Second Hospital of Hebei Medical University. All participants had signed the informed consent.

The inclusion criteria included the following: (1) patients who met the diagnostic criteria of delayed encephalopathy after acute carbon monoxide poisoning (2): ① having a clear history of carbon monoxide (CO) poisoning; ② disappearance of acute poisoning symptoms after treatment; ③ symptoms including cognitive and psychosis dysfunction, mental disorder, extrapyramidal symptoms, agnosia, temporary deafness, visual field changes, temporary decline in spatial recognition ability and vegetative coma that occurred within 2–60 days after a complete rehabilitation; ④ CT/MRI showing no specific changes or extensive white matter lesions. (3) Patients aged between 18 and 80 years old; (4) first onset; (5) patients or their family members who signed the consent form.

Exclusion criteria included: (1) patients who had a history of cerebral infarction, cerebral haemorrhage or trauma; (2) patients who had cognitive impairment or could not take care of themselves before onset; (3) patients who had a history of psychosis; (4) patients or their family members who did not agree to participate.

All subjects were randomly divided into two groups according to the random number table: a treatment group (*n* = 20) and a control group (*n* = 20). There were no significant differences between the two groups in terms of age, gender, education level, latent phase (Time from acute carbon monoxide poisoning to delayed encephalopathy) and visit time (Time between the occurrence of delayed encephalopathy after acute carbon monoxide poisoning and the first evaluation) (Table 1).

**Table 1:** Comparison of the general information of patients between two groups.

**Groups**	**Number of patients/group**	**Ages (years)**	**Gender**		**Latent phase (days)**	**Visit time (days)**	**Education level**		
			**Male**	**Female**			**Illiteracy**	**Primary school**	**Secondary school and above**
Treatment group	20	59 ± 10	9	11	23 ± 9	10 ± 8	5	7	8
Control group	20	62 ± 8	12	8	19 ± 7	8 ± 6	3	10	7

*latent phase: time from acute carbon monoxide poisoning to delayed encephalopathy*.

*visit time: time between the occurrence of delayed encephalopathy after acute carbon monoxide poisoning and the first evaluation*.

### Therapeutic Approach

Both groups of patients underwent hyperbaric oxygen therapy, conventional individualised rehabilitation therapy and received medications (hormones, neurotrophic drugs, anticoagulants, free radicals scavenging, and intracranial hypotensive drugs) for the improvement of microcirculation and nutritional nerves; the treatment group was additionally treated with transcranial direct current stimulation. Patients underwent 10 treatment sessions; one session per day, six times a week, excluding Sunday. One course of treatment lasted for 10 days, and the therapeutic effect was evaluated after three courses. All patients were treated alone. There is no interaction between patients.

#### Hyperbaric Oxygen Therapy

The medical three-compartment and seven-door air compression chamber (YC3200/0.3-22) produced by Yantai Ice Wheel Factory was used for treatment. The time for compression and decompression was 25 min. The treatment pressure was set to 0.1 MPa. During the pressure stabilisation period, pure oxygen was inhaled through a mask for 30 min; this was conducted over two sessions, with an interval of 10 min to allow for the inhalation of the air in the compartment. The total time for one treatment was 120 min.

#### Conventional Rehabilitation Therapy

Routine, individualised symptomatic rehabilitation, including physical recovery, occupational therapy, speech training, psychological counselling and cognitive training, was also performed. Each treatment lasted 30–40 min.

#### Transcranial Direct Current Stimulation

For the treatment group, transcranial direct current stimulation (model number: IS200, Sichuan Intelligent Electronics Industry Co., Ltd.) was used. The surface electrodes were composed of gelatine sponge, with an area of 7 cm × 5 cm and a battery-driven constant current intelligent stimulator. The intensity of the stimulus current was adjusted to 1–2 mA, depending on the tolerance of the patient. The time was set to 20 min/time. Anode electrodes were placed on the projection position of the left dorsolateral prefrontal cortex on the body of the patient, while cathode electrodes were placed on the right orbital or right shoulder as reference electrodes.

### Assessment Approach

The Mini-Mental State Examination (MMSE) score (15) and the latency and amplitude of endogenous event-related evoked potential P300 (16) were used for cognitive function assessment. Conduct P300 detection in a quiet environment. The patient takes a sitting or supine position, closes his eyes, focuses his attention and relaxes his whole body. Place electrodes according to the international 10/20 system for recording ordinary EEG, place the recording electrode at the CZ point on the skull top, place the reference electrode at the inner side of the earlobe on both sides (A1 point on the left ear and A2 point on the right ear), and place the grounding electrode at the FPZ point in the middle of the forehead. Explain the test process and requirements to the subjects before the test, and conduct the pre-test at least once. During the test, auditory Oddball mode is selected for sound stimulation, which is target stimulation sound and non-target stimulation sound. The two stimulation sounds are superimposed for 100 times, in which the probability of target stimulation sound is 20% and the probability of non-target stimulation sound is 80%. The subjects are required to report the times of target stimulation after the test. Finally, the latency and amplitude of P300 were measured. The activity of daily living was assessed using the Barthel Index score (17). The cognitive function and activity of daily living were assessed in both groups before and after three courses of treatment.

### Statistical Analysis

Statistical analysis was performed using SPSS 21.8 software. The measurement data were represented as mean ± standard deviation. The categorical variables were expressed as frequency [percentage (%)]. For two comparisons, each value was compared by *t*-test when each datum conformed to normal distribution, while the non-normally distributed continuous data were compared using non-parametric tests. The counting data were tested by chi-square test. A value of *P* < 0.05 was considered statistically significant.

## Results

### General Information of Patients

No significant differences in age, gender, educational level, latent phase and visit time were found between the control and treatment groups (*P* < 0.05) (Table 1).

### Comparison of Mini-Mental State Examination and Barthel Index Scores Between Groups

In both groups, significantly higher Mini-Mental State Examination and Barthel Index scores were observed after treatment compared to those before treatment (*P* < 0.05); however, scores in the treatment group were significantly higher compared to the control group (*P* < 0.05) (Table 2).

**Table 2:** Comparison of MMSE, BI scores, P300 latency, and amplitude between two groups before and after treatment ($\bar{x}$±s, *n* = 20).

**Group**	**MMSE score**		**BI score**		**P300 latency (ms)**		**P300 amplitude (μV)**	
	**Before treatment**	**After treatment**	**Before treatment**	**After treatment**	**Before treatment**	**After treatment**	**Before treatment**	**After treatment**
Treatment group	6 ± 5	13 ± 7^a^^b^	29 ± 16	54 ± 17^a^^b^	372 ± 10	342 ± 29^a^^b^	4.3 ± 2.3	7.1 ± 3.3^a^^b^
Control group	4.8 ± 3.7	9 ± 5^a^	20 ± 15	43 ± 15^a^	379 ± 14	363 ± 17^a^	3.3 ± 1.8	5.1 ± 2.7^a^

*MMSE, Mini-mental State Examination; BI, Barthel Index. Compared with before treatment*,

a *P < 0.05; Compared with the control group*,

b *P < 0.05*.

### Comparison of P300 Latency and Amplitude Between Groups

After treatment, there was a reduction in P300 latency and an increase in P300 amplitude in both groups compared to before treatment (*P* < 0.05); however, the reduction in latency and increase in amplitude observed in the treatment group were significantly greater compared to the control group (*P* < 0.05) (Table 2).

In addition, no significant differences were detected in Mini-Mental State Examination and Barthel Index scores, or in P300 latency and amplitude, between the two groups before treatment (*P* > 0.05).

## Discussion

The outcomes of this study presented that Mini-Mental State Examination and Barthel Index scores were significantly higher after treatment when compared to those before treatment, the scores in the treatment group were also significantly higher than the control group. After treatment, there was a reduction in P300 latency and an increase in P300 amplitude in both groups compared to before treatment, the reduction in latency and increase in amplitude observed in the treatment group were significantly greater compared to the control group.

Delayed encephalopathy after acute carbon monoxide poisoning is characterised by a recurrence of neurological or psychiatric symptoms, which frequently cause diffuse brain damage in patients; however, the exact pathogenesis of delayed encephalopathy after acute carbon monoxide poisoning still remains unclear. At present, aside from medications, hyperbaric oxygen therapy and various rehabilitation methods have been applied in the clinical treatment of delayed encephalopathy after acute carbon monoxide poisoning (18–20). Previous studies have shown transcranial direct current stimulation to be a very effective treatment approach for post-stroke cognitive impairment (21), Parkinson's disease (8) and Alzheimer's disease (9). Bennabi et al. (22) have found that when synaptic activity occurs, transcranial direct current stimulation stimulate of the cerebral cortex could cause the accumulation of intracellular Ca^2+^ in neuronal cells of synaptic terminals, presynaptic neurons and postsynaptic neurons; this can, therefore, lead to long-term potentiation and long-term inhibition, both of which are currently recognised as the cellular basis of learning and memory (23). By increasing regional cerebral blood flow and promoting cerebral circulation, transcranial direct current stimulation also regulates cognitive function. Merzagora et al. have found that anodic transcranial direct current stimulation has the potential to prolong the relaxation of the cerebrovascular system and increase the cerebral blood flow (24), while the increased blood supply in the dorsolateral prefrontal cortex dorsolateral prefrontal cortex improves the cognitive function (25). After acute carbon monoxide poisoning, vascular injury and cerebral circulation disturbance could cause brain edoema and secondary microcirculatory disturbance. These, in turn, can lead to ischemic softening in the medial part of the globus pallidus, which has a lower blood supply, and to extensive demyelination changes in the white matter of the brain, eventually resulting in cognitive impairment of memory, learning ability, attention and motor dysfunction in delayed encephalopathy after acute carbon monoxide poisoning patients (2).

This study used a randomised controlled trial. The results showed that the MMSE and BI scores in the treatment group were significantly increased after treatment, and the P300 latency were significantly shortened and P300 amplitude were significantly decreased. Therefore, it is believed that tDCS combined with HBOT is helpful to improve the cognitive function and ADL of DEACMP patients. The stimulating sites, the polarity of the stimulus electrode and the stimulus parameters have a great impact on the therapeutic efficacy of transcranial direct current stimulation. Based on previous studies (26–29) and our preliminary data, the stimulating sites selected in this experiment were all left dorsolateral prefrontal cortex with a stimulating power of 1–2 mA and a stimulating time of 20 min. The curative effect was evaluated after three courses of continuous treatment; each course consisted of 10 treatments, which were performed each day of the week, except Sunday. The use of transcranial direct current stimulation resulted in a significantly higher Mini-Mental State Examination score (13 ± 7 points) compared to before treatment (6 ± 5 points), and also when compared to the control group (9 ± 5 points); the most significant improvements were observed in memory, orientation and naming ability. In addition, during the course of the treatment, no discomfort occurred in other patients, except for the itching of the scalp at the stimulating site, which was observed in some patients.

A kind of endogenous cognitive potential, P300 is mainly used in the evaluation and research of cognitive impairment (30). Braverman et al. (31) have suggested that the prolongation of P300 latency could be used to predict early clinical cognitive impairment. In the present study, we found that the Mini-Mental State Examination score and the P300 amplitude of the treatment group were increased, while the latency was reduced compared with the control group, suggesting that transcranial direct current stimulation could improve the cognitive function of patients. The mechanism might be related to the increase of synaptic plasticity induced long-term potentiation and long-term depression by anodic transcranial direct current stimulation, and the promotion of cerebral circulation caused by increased regional cerebral blood flow.

Cognitive function is closely associated with activity of daily living. Yuan et al. (32) have found that cognitive training has the potential improve the cognitive function of delayed encephalopathy after acute carbon monoxide poisoning patients, including their activity of daily living. Researches have confirmed that cognitive impairment could affect activity of daily living in stroke patients (33) and craniocerebral injury patients (34). The results of this study showed that the Barthel Index score of the treatment group was significantly higher than that of the control group, which suggests that the improvement of activity of daily living was more significant.

In addition, the mechanism of DEACMP has the following assumptions. Recent studies have shown that at the molecular level, affective disorder and suicide behaviour are related to structural and synaptic plasticity disorders. Small non-coding RNAs (ncRNAs), especially microRNAs (miRNAs), play an important role in the translation and regulation of synapses (35). There are also studies suggesting that an imbalance in glutamatergic neurotransmission may lead to increased levels of N-methyl-D-aspartate (NMDA) agonists, thereby enhancing excitatory activity in most brain circuits involved in major depression (36). MicroRNAs and NMDA may be involved in the pathogenesis of DEACMP.

This study has some limitations; the sample size was too small, and the observation time was too short. A larger number of samples are, therefore, required to further compare and optimise the stimulus parameters and therapeutic effects. The later effects of cognitive function and quality of life of the patients after discharge will be followed up and additionally reported. In addition, this study mainly described the clinical efficacy with less research on related mechanisms. Subsequent studies will focus on the pathogenesis of DEACMP.

In this study, a randomised controlled trial was conducted. A total of 20 delayed encephalopathy after acute carbon monoxide poisoning patients were enrolled in both the treatment group and the control group, respectively. The control group was treated with hyperbaric oxygen therapy, drug therapy and conventional rehabilitation therapy; the treatment group was treated with the combination of these therapies and transcranial direct current stimulation, once a day, 6 days a week, and 10 times per course. After three courses of treatment, the cognition and activity of daily living abilities of patients both in the control group and the treatment group were significantly improved; the improvement of patients in the treatment group, however, was more significant. The combination of hyperbaric oxygen therapy, drug therapy and conventional rehabilitation therapy with transcranial direct current stimulation treatment maybe more effective at improving the cognitive function and activity of daily living of delayed encephalopathy after acute carbon monoxide poisoning patients. Therefore, adding transcranial direct current stimulation to hyperbaric oxygen therapy maybe worthy of application for patients with delayed encephalopathy after carbon monoxide poisoning in clinical practise.

## Data Availability Statement

The original contributions presented in the study are included in the article/supplementary material, further inquiries can be directed to the corresponding author/s.

## Ethics Statement

The studies involving human participants were reviewed and approved by Ethics Committee of the Second Hospital of Hebei Medical University. The patients/participants provided their written informed consent to participate in this study.

## Author Contributions

HC, XT, LZ, and HL: conception and design of the research. ZL, CL, HC, and XT: acquisition of data. ML, LC, and LZ: analysis and interpretation of the data. HC, XT, and ML: statistical analysis. HC, XT, and ZL: writing of the manuscript. HL: critical revision of the manuscript for intellectual content. All authors contributed to the article and approved the submitted version.

## Conflict of Interest

The authors declare that the research was conducted in the absence of any commercial or financial relationships that could be construed as a potential conflict of interest.

## Publisher's Note

All claims expressed in this article are solely those of the authors and do not necessarily represent those of their affiliated organizations, or those of the publisher, the editors and the reviewers. Any product that may be evaluated in this article, or claim that may be made by its manufacturer, is not guaranteed or endorsed by the publisher.

## References

[1] Niu FY Zhao JY. Advances in delayed encephalopathy induced by acute carbon monoxide. Chin J Indust Hygiene Occup Dis. (2001) 19:397–8. 10.3760/cma.j.issn.1001-9391.2001.05.043

[2] Tao HY Jiang GD Lin F. Clinical application of hyperbaric oxygen. Shanghai: Second Military Medical University Press (2015). p. 137–8.

[3] Cobb N Etzel RA. Unintentional carbon monoxide-related deaths in the United States, 1979 through 1988. JAMA. (1991) 266:659–63. 10.1001/jama.1991.03470050059023 1712865

[4] Zhang J Guo Y Li W Li G Chen Y. The efficacy of N-butylphthalide and dexamethasone combined with hyperbaric oxygen on delayed encephalopathy after acute carbon monoxide poisoning. Drug Des Devel Ther. (2020) 14:1333–9. 10.2147/DDDT.S217010 32308366 PMC7135188

[5] Huang YQ Peng ZR Huang FL Yang AL. Mechanism of delayed encephalopathy after acute carbon monoxide poisoning. Neural Regen Res. (2020) 15:2286–95. 10.4103/1673-5374.284995 32594050 PMC7749483

[6] Lefaucheur JP. Methods of therapeutic cortical stimulation. Neurophysiol Clin. (2009) 39:1–14. 10.1016/j.neucli.2008.11.001 19268842

[7] Fan JJ Xu QL Guo L Wang Q. Application of transcranial direct current stimulation in rehabilitation after stroke. J Clin Neurol. (2016) 29:76–7.

[8] Manenti R Brambilla M Benussi A Rosini S Cobelli C Ferrari C. Mild cognitive impairment in Parkinson's disease is improved by transcranial direct current stimulation combined with physical therapy. Mov Disord. (2016) 31:715–24. 10.1002/mds.26561 26880536

[9] Elder GJ Taylor JP. Transcranial magnetic stimulation and transcranial direct current stimulation: treatments for cognitive and neuropsychiatric symptoms inthe neurodegenerative dementias. Alzheimers Res Ther. (2014) 6:74. 10.1186/s13195-014-0074-1 25478032 PMC4255638

[10] Tekturka P Erdogan ET Kurt A Vanli-Yavuz EN Ekizoglu E Kocagoncu E. The effect of transcranial direct current stimulation on seizure frequency of patients with temporal lobe epilepsy with hippocampal sclerosis. Clin Neurol Neurosurg. (2016) 149:27–32. 10.1016/j.clineuro.2016.07.014 27450765

[11] Boggio PS Zaghi S Fregni F. Modulation of emotions associated with images of human pain using anodal transcranial direct current stimulation (tDCS). Neuropsychologia. (2009) 47:212–7. 10.1016/j.neuropsychologia.2008.07.022 18725237

[12] Salehinejad MA Ghanavai E Rostami R Nejati V. Cognitive control dysfunction in emotion dysregulation and psychopathology of major depression (MD): evidence from transcranial brain stimulation of the dorsolateral prefrontal cortex (DLPFC). J Affect Disord. (2017) 210:241–8. 10.1016/j.jad.2016.12.036 28064113

[13] Lefaucheur JP Antal A Ayache SS Benninger DH Brunelin J Cogiamanian F. Evidence-based guidelines on the therapeutic use of transcranial direct current stimulation (tDCS). Clin Neurophysiol. (2017) 128:56–92. 10.1016/j.clinph.2016.10.087 27866120

[14] Sanches C Stengel C Godard J Mertz J Teichmann M Migliaccio R. Past, present, and future of non-invasive brain stimulation approaches to treat cognitive impairment in neurodegenerative diseases: time for a comprehensive critical review. Front Aging Neurosci. (2021) 12:578339. 10.3389/fnagi.2020.578339 33551785 PMC7854576

[15] Simple intelligence scale (MMSE). Chin J Neurosurg. (2012) 28:1283.

[16] Zhao XD. Event-related potential (continued). J Modern Electrophysiol. (2008) 1:35–40. 10.3969/j.issn.1672-0458.2007.04.014

[17] Min Y Wu YY Yan TB. Validity and reliability of modified Barthel index (simplified Chinese version) scale for assessing activities of daily living in stroke patients. Chin J Phys Med Rehabil. (2008) 30:185–9. 10.3321/j.issn:0254-1424.2008.03.010

[18] Liu YL Zhang HX Yu QH Xue LB. Observation of the therapeutic effect of hyperbaric oxygen on delayed encephalopathy caused by carbon monoxide poisoning. Chin J Phys Med Rehabil. (2015) 37:201–4. 10.3760/cma.j.issn.0254-1424.2015.03.01324308190

[19] Oric V Oren DA Wolkenberg FA Kravitz RE. Carbon monoxide poisoning and treatment with hyperbaric oxygen in the subacute phase. J Neurol Neurosurg Psychiatry. (1998) 65:245–7. 10.1136/jnnp.65.2.245 9703180 PMC2170185

[20] Zhao SH Li WR. Clinical observation of butylphthalide soft capsule in the treatment of delayed encephalopathy caused by carbon monoxide poisoning. Chin J Neuroimmunol Neurol. (2017) 24:349–52. 10.3969/j.issn.1006-2963.2017.05.009

[21] Yun GJ Chun MH Kim BR. The effects of transcranialdirect-current stimulation on cognition in stroke patients. J Stroke. (2015) 17:354–8. 10.5853/jos.2015.17.3.354 26438001 PMC4635724

[22] Bennabi D Pedron S Haffen E Monnin J Peterschmitt Y Van Waes V. Transcranial direct current stimulation for memory enhancement: from clinical research to animal models. Front Syst Neurosci. (2014) 8:159. 10.3389/fnsys.2014.00159 25237299 PMC4154388

[23] Sigurdsson T Doyere V Cain CK LeDoux JE. Long-term potentiation in the amygdala: a cellular mechanism of fear learning and memory. Neuropharmacology. (2007) 52:215–27. 10.1016/j.neuropharm.2006.06.022 16919687

[24] Merzagora AC Foffani G Panyavin I Mordillo-Mateos L Aguilar J Onaral B. Prefrontal hemodynamic changes produced by anodal direct current stimulation. Neuroimage. (2010) 49:2304–10. 10.1016/j.neuroimage.2009.10.044 19853048

[25] Elder GJ Ashcroft J Morgan KS Kulsum MU Banerjee R Chatterjee P. Transcranial direct current stimulation in Parkinson's disease dementia: a randomised double-blind crossover trial. Brain Stimul. (2017) 10:1150–1. 10.1016/j.brs.2017.07.012 28802804

[26] Boggio PS Ferrucci R Mameli F Martins D Martins O Vergari M. Prolonged visual memory enhancement after direct current stimulation in Alzheimer's disease. Brain Stimul. (2012) 5:223–30. 10.1016/j.brs.2011.06.006 21840288

[27] Meinzer M Lindenberg R Phan MT Ulm L Volk C Flöel A. Transcranial di rect current stimulation in mild cognitive impairment: behavioral effects and neural mechanisms. Alzheimers Dement. (2015) 11:1032–40. 10.1016/j.jalz.2014.07.159 25449530

[28] Cotelli M Manenti R Brambilla M Petesi M Rosini S Ferrari C. Anodal tDCS during face-name associations memory training in Alzheimer's patients. Front Aging Neurosci. (2014) 6:38. 10.3389/fnagi.2014.00038 24678298 PMC3958642

[29] Elder GJ Firbank MJ Kumar H Chatterjee P Chakraborty T Dutt A. Effects of transcranial direct current stimulation upon attention and visuoperceptual function in Lewy body dementia: a preliminary study. Int Psychogeriatr. (2015) 28:1–7. 10.1017/S1041610215001180 26250473 PMC4720143

[30] Zhu H Zhang JJ. Clinical application of ERP P3a and P3b in cognitive function. Chin J Clin Neurosci. (2008) 16:94–8. 10.3969/j.issn.1008-0678.2008.01.023

[31] Braverman ER Chen TJ Schoolfield J Martinez-Pons M Arcuri V Varshavskiy M. Delayed P300 latency correlates with abnormal test of variables of attention (TOVA) in adults and predicts early cognitive decline in a clinical setting. Adv Ther. (2006) 23:582–600. 10.1007/BF02850047 17050501

[32] Yuan P Wang LP. Observation on the effect of cognitive rehabilitation on delayed encephalopathy dementia patients caused by acute carbon monoxide poisoning. Chin J Phys Med Rehabil. (2012) 34:386–7. 10.3760/cma.j.issn.0254-1424.2012.05.020

[33] Naruishi K Kunita A Kubo K Nagata T Takashiba S Adachi S. Predictrs of improved functional outcome in elderly inpatients after rehabilitation:a retrospective study. Clin Interv Aging. (2014) 9:2133–41. 10.2147/CIA.S73388 25584025 PMC4264602

[34] Dikmen SS Corrigan JD Levin HS Machamer J Stiers W Weisskopf MG. Cognitive outcome following traumatic brain injury. J Head Trauma Rehabil. (2009) 24:430–8. 10.1097/HTR.0b013e3181c133e9 19940676

[35] Serafini G Pompili M Innamorati M Giordano G Montebovi F Sher L. The role of microRNAs in synaptic plasticity, major affective disorders and suicidal behavior. Neurosci Res. (2012) 73:179–90. 10.1016/j.neures.2012.04.001 22521503

[36] Serafini G Pompili M Innamorati M Dwivedi Y Brahmachari G Girardi P. Pharmacological properties of glutamatergic drugs targeting NMDA receptors and their application in major depression. Curr Pharm Des. (2013) 19:1898–922. 10.2174/13816128113199990293 23173582

